# Diffusion tensor imaging in cubital tunnel syndrome

**DOI:** 10.1038/s41598-021-94211-7

**Published:** 2021-07-22

**Authors:** Timothy T. Griffiths, Robert Flather, Irvin Teh, Hamied A. Haroon, David Shelley, Sven Plein, Grainne Bourke, Ryckie G. Wade

**Affiliations:** 1 Leeds Institute for Medical Research, University of Leeds, Leeds, UK; 2 Department of Plastic, Reconstructive and Hand Surgery, Leeds Teaching Hospitals Trust, Leeds, UK; 3 Leeds Institute for Cardiovascular and Metabolic Medicine, University of Leeds, Leeds, UK; 4 The Advanced Imaging Centre, Leeds Teaching Hospitals Trust, Leeds, UK; 5 Division of Neuroscience and Experimental Psychology, The University of Manchester, Manchester, UK

**Keywords:** Neurological disorders, Translational research

## Abstract

Cubital tunnel syndrome (CuTS) is the 2nd most common compressive neuropathy. To improve both diagnosis and the selection of patients for surgery, there is a pressing need to develop a reliable and objective test of ulnar nerve ‘health’. Diffusion tensor imaging (DTI) characterises tissue microstructure and may identify differences in the normal ulnar from those affected by CuTS. The aim of this study was to compare the DTI metrics from the ulnar nerves of healthy (asymptomatic) adults and patients with CuTS awaiting surgery. DTI was acquired at 3.0 T using single-shot echo-planar imaging (55 axial slices, 3 mm thick, 1.5 mm^2^ in-plane) with 30 diffusion sensitising gradient directions, a b-value of 800 s/mm^2^ and 4 signal averages. The sequence was repeated with the phase-encoding direction reversed. Data were combined and corrected using the FMRIB Software Library (FSL) and reconstructed using generalized q-sampling imaging in DSI Studio. Throughout the length of the ulnar nerve, the fractional anisotropy (FA), quantitative anisotropy (QA), mean diffusivity (MD), axial diffusivity (AD) and radial diffusivity (RD) were extracted, then compared using mixed-effects linear regression. Thirteen healthy controls (8 males, 5 females) and 8 patients with CuTS (6 males, 2 females) completed the study. Throughout the length of the ulnar nerve, diffusion was more isotropic in patients with CuTS. Overall, patients with CuTS had a 6% lower FA than controls, with the largest difference observed proximal to the cubital tunnel (mean difference 0.087 [95% CI 0.035, 0.141]). Patients with CuTS also had a higher RD than controls, with the largest disparity observed within the forearm (mean difference 0.252 × 10^–4^ mm^2^/s [95% CI 0.085 × 10^–4^, 0.419 × 10^–4^]). There were no significant differences between patients and controls in QA, MD or AD. Throughout the length of the ulnar nerve, the fractional anisotropy and radial diffusivity in patients with CuTS are different to healthy controls. These findings suggest that DTI may provide an objective assessment of the ulnar nerve and potentially, improve the management of CuTS.

## Introduction

Cubital tunnel syndrome (CuTS) is the 2nd most common compressive neuropathy, affecting 36 per 100,000 person years^1^ or 6% of the population^2^. Chronic compression leads to distortion of the axonal architecture and demyelination, followed by poor remyelination. Fibrosis of the perineurial and epineurial connective tissue, and its vasculature, occur simultaneously^3,4^. Surgical decompression is the most effective treatment and approximately 15,000 people per annum undergo surgical decompression in the UK^5^ and USA^6^.

Patients present with a mixture of sensory and motor symptoms. The sensory symptoms include cutaneous dysaesthesias (such as pins and needles), hypoaesthesia or anaesthesia in the little and ring fingers, alongside pain. The motor symptoms can include weakness and dyspraxia of the hand. Provocative tests are few and unreliable^7,8^. Despite normal electrodiagnostic tests, surgery is still offered to symptomatic patients’^9^. Moreover, surgery is unbeneficial in 13% of patients and 3% develop serious complications (e.g., infection or haematoma) requiring reoperation^10^. This suggests that clinicians lack a reliable, reproducible and objective test to select patients for surgery. Furthermore, no currently available tests can provide an objective assessment of the ‘health’ of the ulnar nerve postoperatively^11^.

Diffusion magnetic resonance imaging (dMRI) characterises tissue microstructure and provides reproducible^12–15^ proxy measures of nerve ‘health’ which are sensitive to myelination, axon diameter, fibre density and organisation^16–20^. Diffusion tensor imaging (DTI) is a type of dMRI which typically generates the following metrics: fractional anisotropy (FA), mean diffusivity (MD), axial diffusivity (AD) and radial diffusivity (RD). FA is a scalar value between zero and one, whereby an FA of zero implies isotropic diffusion of water molecules within a voxel, whilst a FA nearing one implies diffusion which is restricted to a single axis (bidirectional diffusion along the length of the nerve). MD describes the average molecular diffusion rate within the voxel, whilst AD describes diffusion in the long axis and RD represents diffusion perpendicular to the long axis. Based on the Fourier transform relation between the dMRI signals and the underlying diffusion displacement, quantitative anisotropy (QA) can be estimated using generalised q-sampling imaging (GQI)^21^. As QA scales with spin density and the dMRI signals, it has arbitrary units from 0 upwards which cannot be compared between subjects, therefore QA is scaled to a maximum of 1 to yield normalised QA (nQA). Several studies have reported the findings of dMRI metrics from the ulnar nerve in asymptomatic adults^22–27^. These metrics may be more reliable than nerve conduction and electromyography^24,26^. To-date, only one study has reported on DTI in CuTS although this article only summarised the data in graphical format, omitting to report the point estimates and variance any diffusion metrics (e.g. FA or any measures of diffusivity) from the ulnar nerve which hinders the interpretation and limits the external validity^24^.

This proof-of-concept study aimed to determine differences in DTI parameters of the ulnar nerve between healthy volunteers and patients with CuTS. This may help to determine if DTI could play a role in the clinical management of the condition.

## Methods

This prospective cross-sectional study was designed and reported in accordance with the STROBE and STARD guidance, taking into account the domains of the QUADAS-2^28^ and PRISMA-DTA^29^ tools. Approval was provided by the National Health Research Authority (ID 19/NW/0324) and written informed consent was obtained from all participants.

### Recruitment

Consecutive patients with a recent diagnosis of CuTS who were scheduled for decompressive surgery in our institution were recruited between July 2019 and March 2020. The clinical diagnosis of CuTS was made by a hand surgeon with subspecialist interest in peripheral nerve surgery, supported by the use of electrodiagnostics (neurophysiology) and having excluded proximal pathology. No additional imaging (e.g., ultrasound) was performed. We excluded those with a concurrent or previous peripheral neuropathy, non-MRI safe active implants, metallic implants near the elbow, claustrophobia and those unable to remain still for scanning (e.g., due to pain, dystonia, etc.). Controls (> 18 years old) were recruited with identical exclusion criteria.

### Image acquisition

DTI data were acquired at a field strength of 3.0 T (T) using a Siemens Magnetom Prisma (Siemens Healthcare Limited, Erlangen, Germany) MRI system and single-shot echo-planar imaging (ssEPI). Participants were scanned prone, with the shoulder flexed and elbow straight. The elbow was positioned as close to isocentre of the magnet as comfortably possible. A 4-channel flexible coil was wrapped around the elbow and secured with strapping. Fifty-five axial slices of 3 mm thickness were acquired, at an in-plane resolution of 1.5 mm^2^^11^. The field-of-view was reduced to 192 × 165 × 78 mm using ZOOMit (TimTX TrueShape) with TrueForm b1 shim. We applied 30 non-collinear monopolar diffusion sensitising gradient directions using a Jones scheme^30^ with the following parameters: b-value 800 s/mm^2^, 16 interleaved b0s, TE 74 ms, TR 7800 ms, echo spacing 0.97 ms, echo train length 445 ms, GRAPPA off, 6/8 partial Fourier, receiver bandwidth 1184 Hz, distortion correction off and strong fat saturation. Four signal averages were acquired. The total acquisition time was 17 min 50 s. The sequence was repeated with the (right-to-left) phase-encoding direction reversed. This was supplemented by a T2-weighted TSE of identical geometry and resolution, with TE 69 ms and TR 9790 ms (3 min 42 s).

### Pre-processing

**Figure 1:**
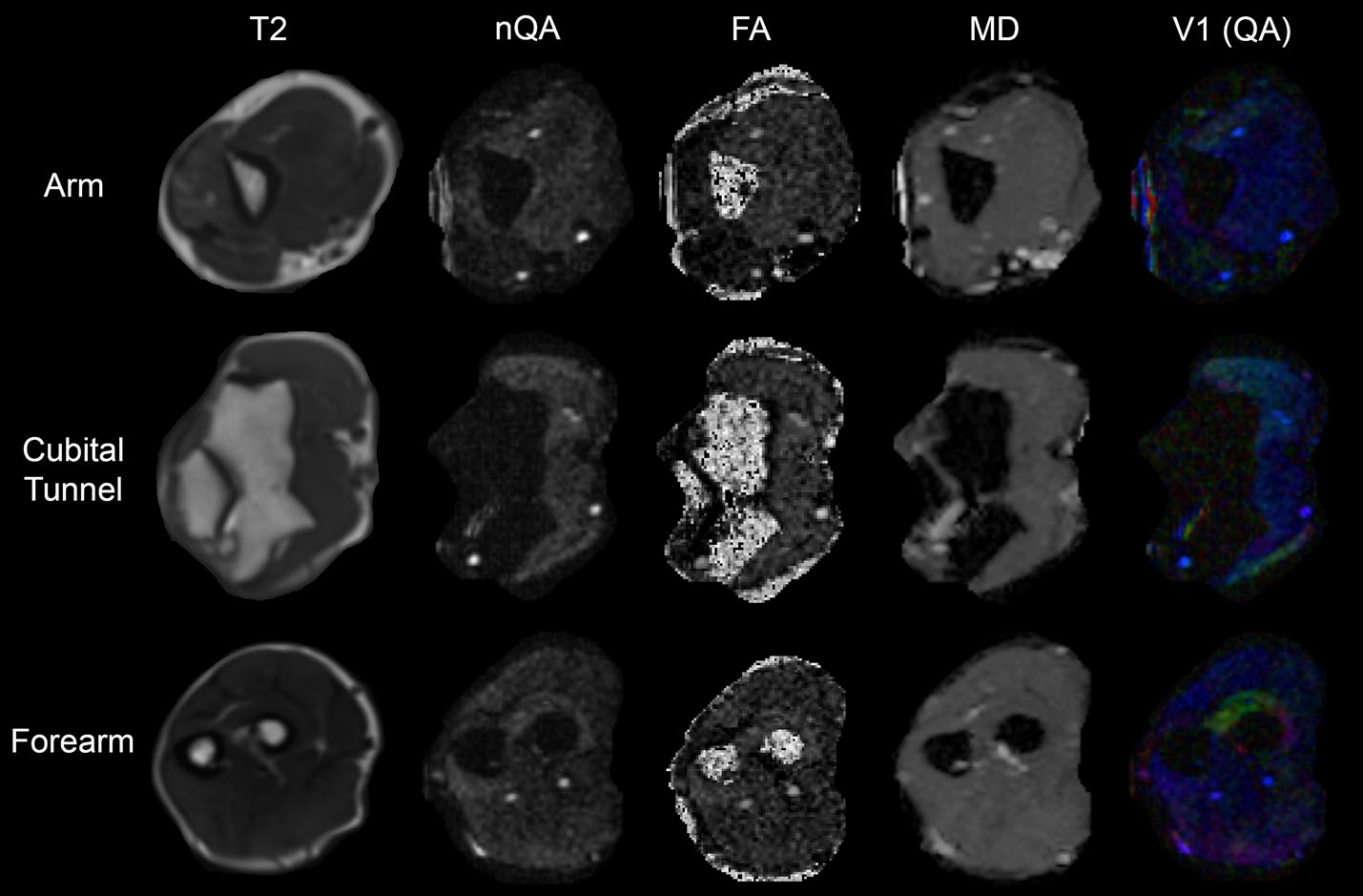
Data derived from a healthy control. The rows show data from the arm, cubital tunnel and forearm. The columns contain T2-weighted scans, and corresponding maps of normalised quantitative anisotropy (nQA), fractional anisotropy (FA), mean diffusivity (MD) and the principal eigenvector (v1) with the colours red, green and blue representing diffusion in x, y and z directions, and the intensity scaled by quantitative anisotropy (QA).

The FMRIB Software Library (FSL) was used to pre-process datasets^31^. Binary masks were made using the BET tool. TOPUP was used (with no subsampling) to correct for susceptibility artefacts. Volumes acquired with opposing phase-encoding directions were combined and corrected for artefacts of motion and eddy-currents using EDDY, with *resamp* = *lsr* (linear least-squares resampling), *repol* and *fep* enabled. The corrected diffusion-weighted dataset and reoriented b-table were then imported to DSI Studio, registered (rigid body) and resampled to the space of T2. Diffusion was quantified using restricted diffusion imaging^32^ and reconstructed using GQI^21^ with a diffusion sampling length ratio of 1.3 (Fig. 1). GQI was chosen because it is a model-free approach, which generates quantitative anisotropy (QA) maps that have substantially better contrast than FA or diffusivity maps for the identification of peripheral nerves (Fig. 1), particularly in proximity to bones and joints. GQI is also readily applicable to numerous different diffusion sampling schemes, the outputs are comparable to more complex q-space methods and it generates a spin-density function which is the closest to reality^21^.

### Regions of interest

After training by RGW (5 years of experience of DTI), TG and RF (6 months of experience of DTI each) placed 3 mm^2^ regions of interest (ROI) on every QA map of every slice, to cover the ulnar nerve. The following metrics were extracted from each ROI: fractional anisotropy (FA), quantitative anisotropy (QA), normalised QA (nQA), radial diffusivity (RD), axial diffusivity (AD) and mean diffusivity (MD).

### Segmentation of the ulnar nerve down the limb

There is no consensus on the exact dimensions nor limits of the cubital tunnel. However, from an anatomical perspective the cubital tunnel extends from the proximal origin of the medial intermuscular septum to the fascial arcade of the two heads of flexor carpi ulnaris. Cadaveric studies have shown that the mean distance from the origin of the medial intermuscular septum to the medial epicondyle is 3.6 cm (maximum 5 cm)^33^ and the mean length of fibro-osseous portion of the cubital tunnel formed by Osborne’s ligament is approximately 3.8 cm (maximum 4.7 cm)^33,34^. Therefore, we classified a 9.9 cm section (33 axial slices of 3mm) centred on the radiohumeral joint, as ‘within the cubital tunnel’. Measurements proximal to the intermuscular septum were classified as ‘within the arm’. Measurements distal to the cubital tunnel were classified as ‘within the forearm’.

### Analysis

Data were analysed using Stata v15 (StataCop LLC, Texas). The only prior publication on this topic to-date did not provide the exact DTI metrics, or their variances between patients and healthy controls so a power calculation was not possible. Scaled variables approximated the normal distribution so were represented by the mean and standard deviation (SD). To estimate the difference in diffusion metrics between controls and patients, mixed-effects linear modelling was used. The fixed effects were the binary status of the individual (control or patient) and age in years. The random effects varied by the individual (1st) and rater (2nd). The inter-rater agreement was estimated from the residual variance, represented by the intraclass correlation coefficient (ICC) and summarised graphically in a Bland–Altman plot. In line with calls for the abolition of p-values, we have minimise their use and avoided the term “statistical significance”^35,36^, instead focussing on the clinical interpretation in relation to point estimates and their 95% confidence intervals (CI).

### Ethical approval

Approval was gained from the National Research and Ethics Service of the United Kingdom (IRAS project ID 260445, HRA REC reference 19/NW/0324).

## Results

In total, 27 adults were scanned (13 patients awaiting decompressive surgery for cubital tunnel syndrome and 14 controls). One control and 4 patient datasets were excluded due to significant motion artefact. One patient was also excluded after scanning because his symptoms resolved, and surgery was cancelled. Therefore, 8 patients and 13 controls formed the final study cohort. Controls and patients were similar except for age, whereby controls were a mean 5 years younger (95% CI 9, 30; Table 1).

**Table 1:** Baseline characteristics.

Characteristics	Healthy controls (n = 13)	Patients with cubital tunnel syndrome (n = 8)	p-value
Mean age (SD)	28 (6)	49 (16)	< 0.001
**Sex (%)**			
Males	8 (62)	6 (75)	0.656
Females	5 (38)	2 (25)	
Mean height in cm (SD)	173 (8.8)	173 (5.7)	0.952
Mean weight in kg (SD)	75 (18)	81 (9)	0.397
Right handed (%)	11 (58)	8 (100)	0.371
Right limb scanned (%)	9 (70%)	4 (50)	0.646

### Fractional anisotropy

**Figure 2:**
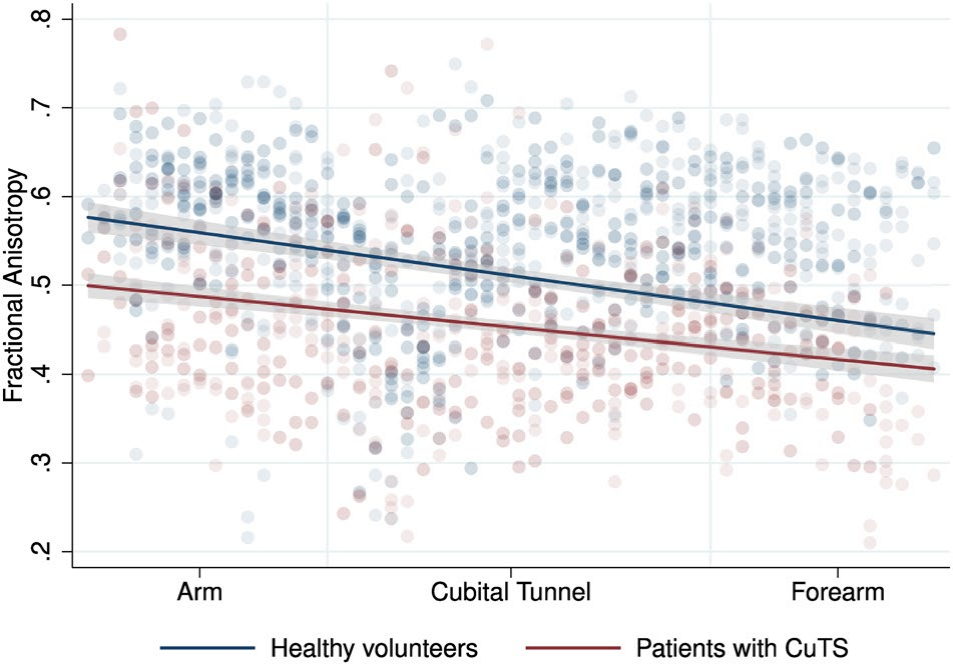
Scatter plot with linear fit (and 95% CI) showing the relationship between fractional anisotropy of the ulnar nerve in volunteers and patients, at different positions within the upper limb.

Patients had a lower FA than controls (mean difference 0.056 [95% CI 0.0004, 0.107]; Fig. 2 and Table 2). The largest disparity was observed in the arm, where the mean difference was 0.087 (95% CI 0.035, 0.141). Within the cubital tunnel the mean difference between controls and patients was 0.054 (95% CI 0.003, 0.105). Fractional anisotropy was not associated with SNR (Supplementary Fig. 1). In the multivariable model, there was a small but independent association between FA and age in both patients (adjusted β − 3.198 × 10^–3^ [95% CI − 3.549 × 10^–3^, − 2.847 × 10^–3^]) and controls (adjusted β − 2.441 × 10^–3^ [95% CI − 3.408 × 10^–3^, − 1.474 × 10^–3^]; Supplementary Fig. 2) which suggests that each decade of life was associated with a ~ 3% reduction in FA.

**Table 2:** Diffusion tensor imaging (DTI) and generalised q-space imaging (GQI) metrics from the ulnar nerve.

Location	Mean (SD)																	
	Fractional anisotropy			Quantitative anisotropy			Normalised quantitative anisotropy			Mean diffusivity (× 10^–3^ mm^2^/s)			Axial diffusivity (× 10^–3^ mm^2^/s)			Radial diffusivity (× 10^–3^ mm^2^/s)		
	Healthy volunteers	Patients	p-value	Healthy volunteers	Patients	p-value	Healthy volunteers	Patients	p-value	Healthy volunteers	Patients	p-value	Healthy volunteers	Patients	p-value	Healthy volunteers	Patients	p-value
Overall	0.511 (0.153)	0.455 (0.099)	< 0.001	0.125 (0.034)	0.123 (0.047)	0.511	0.340 (0.122)	0.345 (0.064)	0.953	1.191 (0.231)	1.310 (0.179)	0.119	2.024 (0.604)	2.008 (0.456)	0.969	0.778 (0.257)	0.958 (0.251)	< 0.001
Within the arm	0.584 (0.047)	0.493 (0.077)	< 0.001	0.131 (0.039)	0.118 (0.044)	0.269	0.360 (0.124)	0.338 (0.101)	0.056	1.119 (0.176)	1.161 (0.092)	0.112	1.977 (0.354)	1.857 (0.160)	0.448	0.690 (0.105)	0.813 (0.111)	< 0.001
Within the cubital tunnel	0.508 (0.092)	0.454 (0.017)	0.036	0.134 (0.037)	0.138 (0.058)	0.278	0.370 (0.142)	0.384 (0.087)	0.873	1.219 (0.221)	1.332 (0.193)	0.057	2.054 (0.368)	2.036 (0.298)	0.910	0.802 (0.156)	0.980 (0.155)	< 0.001
Within the forearm	0.463 (0.182)	0.418 (0.049)	0.437	0.086 (0.058)	0.069 (0.047)	0.641	0.227 (0.142)	0.175 (0.076)	0.420	1.165 (0.447)	1.392 (0.329)	0.067	1.959 (0.767)	2.090 (0.494)	0.463	0.772 (0.294)	1.043 (0.254)	0.003

### Radial diffusivity

**Figure 3:**
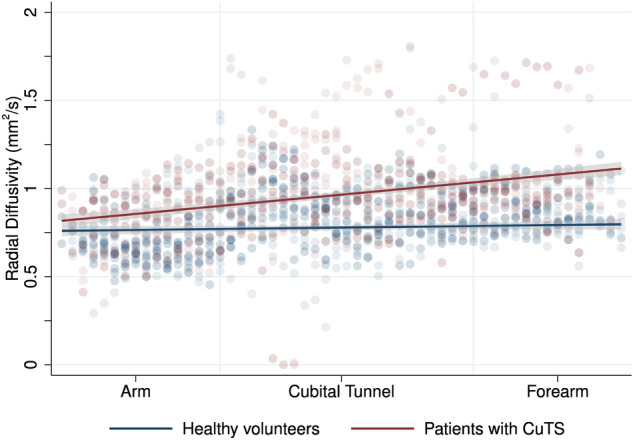
Scatter plot with linear fit (and 95% CI) showing the relationship between radial diffusivity of the ulnar nerve in volunteers and patients, at different positions within the upper limb.

Patients had a higher RD than controls throughout the length of the ulnar nerve (mean difference 0.170 × 10^–4^ mm^2^/s [95% CI 0.144 × 10^–4^, 0.196 × 10^–4^]; Fig. 3 and Table 2). In the multivariable model, this association was independent of age.

The largest disparity was observed in the forearm (mean difference 0.252 × 10^–4^ mm^2^/s [95% CI 0.085 × 10^–4^, 0.419 × 10^–4^]). Within the cubital tunnel the mean difference was 0.169 × 10^–4^ mm^2^/s (95% CI 0.0.84 × 10^–4^, 0.254 × 10^–4^). Within the arm, the mean difference was 0.144 × 10^–4^ mm^2^/s (95% CI 0.082 × 10^–4^, 0.207 × 10^–4^). Radial diffusivity was associated with SNR (β 1.73 × 10^–4^ [95% CI 0.178 × 10^–4^, 3.274 × 10^–4^] p = 0.029; Supplementary Fig. 3).

### Normalised quantitative anisotropy

There was no difference between controls and patients nQA at the level of the arm (Table 2 and Supplementary Fig. 4). As expected, nQA was associated with SNR (β 2.116 × 10^–4^ [95% CI 1.473, 2.759 × 10^–4^] p < 0.001; Supplementary Fig. 5) but not with age (Supplementary Fig. 6).

### Mean diffusivity

There was no difference in MD between controls and patients (Supplementary Fig. 7 and Table 2). MD was not associated with SNR (Supplementary Fig. 8) or age (Supplementary Fig. 9).

### Axial diffusivity

There was no difference in AD between controls and patients (Supplementary Fig. 10 and Table 2). AD was not associated with age (Supplementary Fig. 11) or SNR (Supplementary Fig. 12).

### Inter-rater agreement

There was strong agreement between raters (ICC 0.02 [95% CI 0.002, 0.11]) because the variance in FA due to the person performing the analysis was 0.005 (95% CI 0.002, 0.01; Supplementary Fig. 13).

## Discussion

This study shows that some diffusion tensor imaging metrics of the ulnar nerve in adults with CuTS are different to those of asymptomatic adults. Moreover, these differences appear to manifest throughout the length of the ulnar nerve, not just at the supposed site of compression within the cubital tunnel. As diffusion MRI conveys extra information about tissue microstructure which cannot be obtained through morphological (e.g. T2-weighted) imaging, we believe that diffusion MRI could provide valuable supplementary information in patients with suspected CuTS which may aid management.

Our findings in asymptomatic controls are in agreement with the results of the majority of other studies concerning healthy adults^23–25^. Park et al.^25^ used ssEPI with an identical spatial resolution to us, generating similar DTI metrics (mean FA 0.509) despite fewer than half the number of diffusing sensitising gradient directions, a higher b-value (1200 s/mm^2^) and resultant longer TE (91 ms). Kronlage et al.^23^ generated comparable findings to us using a similar ssEPI protocol, whereby the FA of the ulnar nerve in healthy adults was approximately 0.53. Whilst Breitenseher et al.^24^ did not publish the DTI metrics yielded from 20 healthy adults, their graph summarises the FA of the ulnar nerve at various positions in the limb and was between 0.40 and 0.50. Conversely, Ho^26^ and Zhou^22,27^ yielded DTI metrics which were considerably different to the findings of both our and prior studies^23–25^. Ho^26^ and Zhou^22,27^ used ssEPI sequences with similar b-values (1000 s/mm^2^) and 20 diffusion sensitising gradient directions; however, Ho^26^ used more anisotropic voxels (1.2 × 1.2 mm^2^ in-plane, 4 mm slice thickness) and Zhou^22,27^ worked at higher in-plane resolution (1.0 mm^2^). Both studies yielded estimates of FA which were higher and MD values which were lower than other studies to-date^23–25^. This discrepancy may be explained by low SNR which upwardly biases estimates of FA and underestimates diffusivity^37^. The SNR in Ho’s ssEPI was ~ 11^26^ and Zhou^22,27^ acquired DTI at very high spatial resolution (1 × 1 × 3 mm^3^) with only two repetitions, which is unlikely to recover sufficient SNR to exceed the noise floor. Overall, our healthy adult data appears to be in keeping with the wider literature.

In patients with CuTS, we observed important aberrations in the radial diffusivity and fractional anisotropy of the ulnar nerve. To-date, only one other study^24^ has investigated DTI in patients with CuTS. Breitenseher et al. acquired DTI from 46 patients using ssEPI at 3.0 T, although many important details of the acquisition (e.g. in-plane acceleration or partial Fourier settings, signal averaging, etc.), corrections and tensor fitting methods were not described. They graphed the FA of the ulnar nerve in controls and patients along a 6.4 cm segment of the ulnar nerve but did not publish the exact DTI metrics which hinders the interpretation. Our data builds upon the work of Breitenseher et al.^24^, by addressing important limitations and providing summary estimates of DTI metrics which can be used to inform future studies.

There is no consensus on the definition of CuTS and this is essentially due to the lack of an objective, reliable and repeatable test. Provocative tests^7,8^ and electrodiagnostic studies^9^ are unreliable for diagnosing CuTS and for these reasons, symptomatic patients with normal tests still undergo surgery which renders preoperative testing, ostensibly pointless. Furthermore, surgery is unbeneficial in 13% of patients and 3% develop serious complications which require reoperation^10^. DTI provides proxy measures of nerve health (myelination, axon diameter, fibre density and organisation^16,17^) and as such, DTI metrics may help surgeons to select patients who will benefit from surgical decompression, thus potentially increasing the chance of surgical success and reducing the prevalence of morbidity. Through larger studies, DTI may also provide an objective assessment of nerve recovery after surgery.

All previous studies concerning dMRI of the ulnar nerve performed in-line analysis of diffusion data (i.e. on the scanner workstation) without any form of processing/correction^22–27^. In-line with best practice guidance and the available evidence, we used the FMRIB Software Library (FSL) to correct our diffusion data^31^ but there are no studies which investigate how different software pipelines for the correction of distortions effect the resultant diffusion metrics. Future work should examine what corrections (on the acquisition side as well as within the pre-processing steps) are necessary and how these are best achieved for data derived from the upper limb.

### Limitations

We used the Siemens reduced field-of-view product ZOOMit which deploys a dynamic excitation pulse, potentially permitting a shorter TE (thus better SNR) and reduces distortions. However, ZOOMit was not compatible with our transmit-receive knee coil (which are occasionally used for elbow imaging^23^) and other vendors small field-of-view products (e.g. GE’s FOCUS or Philip’s iZOOM) may perform differently. In our study, age was weakly associated with FA in both patients and controls, but not diffusivity. However, our groups were not balanced in this regard and so the true effect of age on the observed differences in diffusion remains unclear. Futures studies should recruit larger (not ‘age-matched’^38^) samples to better explore the relationship between age and FA. Some experimental factors^39^ can subtly affect diffusion parameter estimates, such as the b-value^40,41^, TE and SNR^37^, the number of diffusion directions whereby coverage of q-space must be balanced against the need for SNR in individual directions^42,43^, the software used to process^44,45^ and reconstruct diffusion data^37^, and the regions of interest used^46^. In an effort to generate reproducible findings, we have followed best practice guidelines or emergent data and detailed our methods. Finally, many questions remain about the relationship between dMRI metrics, function and nerve microstructure. Therefore, future works on dMRI in forearm nerves should seek to incorporate patient-reported outcome measures, other non-invasive proxies of nerve microstructure (such as ultrasound elastography) and if possible, histopathological analysis.

## Conclusions

This proof-of-concept study demonstrated differences in DTI metrics (fractional anisotropy and radial diffusivity) throughout the length of the ulnar nerve, between patients with cubital tunnel syndrome and controls. These findings suggest that DTI may provide an objective assessment of the ulnar nerve and potentially, improve the management of cubital tunnel syndrome.

## Supplementary Information

Supplementary Figures.
