# Supplementary material for: Diffusion tensor imaging in cubital tunnel syndrome
Source: Sci Rep. 2021 Jul 22;11:14982. doi: 10.1038/s41598-021-94211-7 (PMC8298404; doi:10.1038/s41598-021-94211-7)

**eFigure 1.** Scatter plot with linear fit (and 95% CI) showing the relationship between fractional anisotropy and SNR.


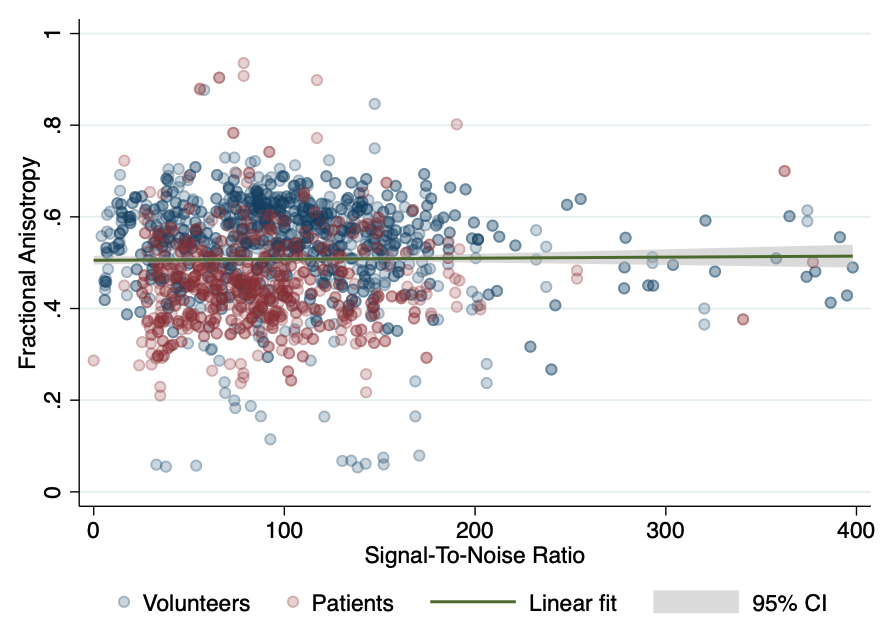


**eFigure 2.** Scatter plot with linear fit (and 95% CI) showing the relationship between fractional anisotropy and age in years.


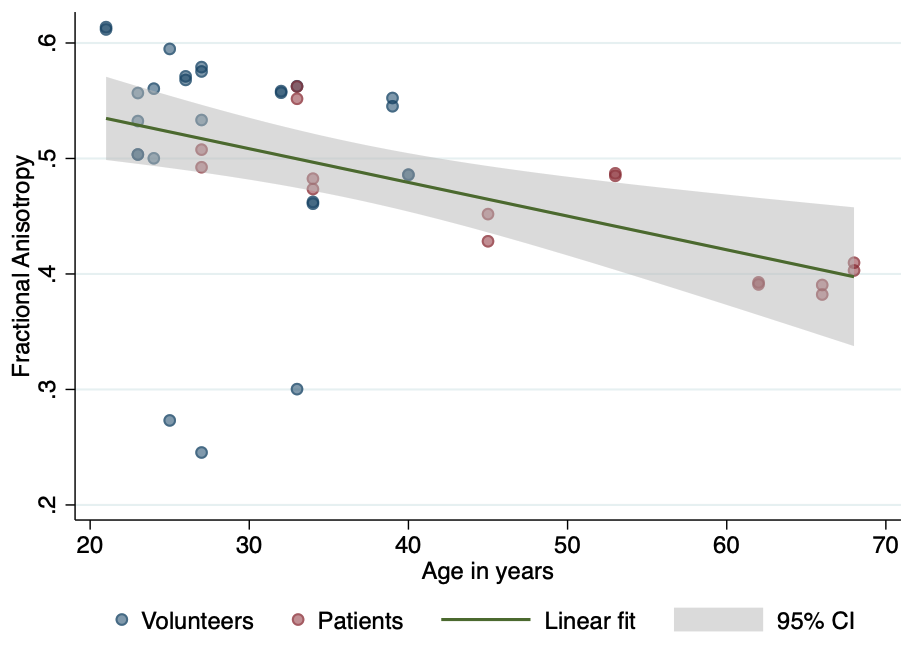


**eFigure 3.** Scatter plot with linear fit (and 95% CI) showing the association between radial diffusivity and SNR.


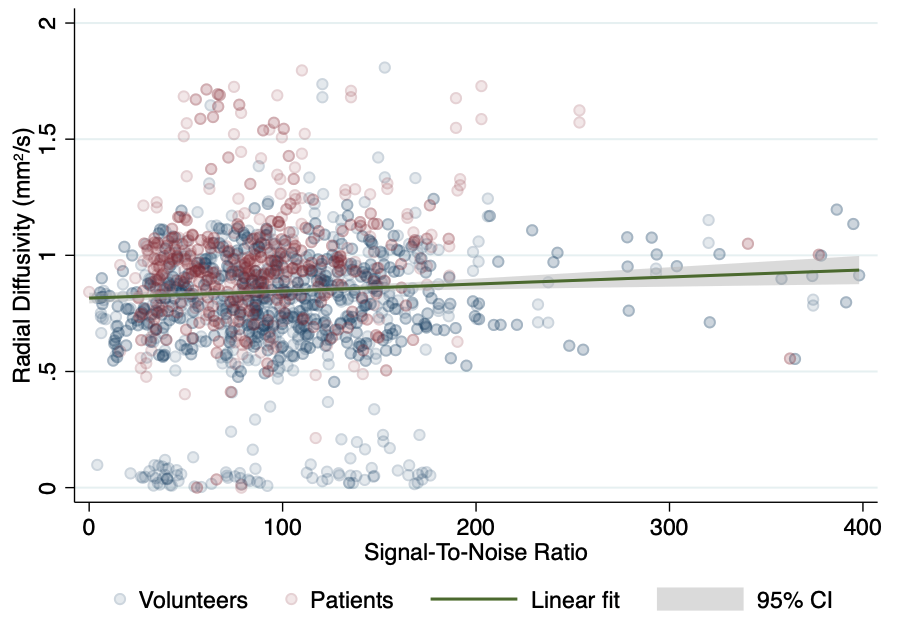


**eFigure 4.** Scatter plot with linear fit (and 95% CI) showing the relationship between normalised quantitative anisotropy of the ulnar nerve in volunteers and patients, at different positions within the upper limb.


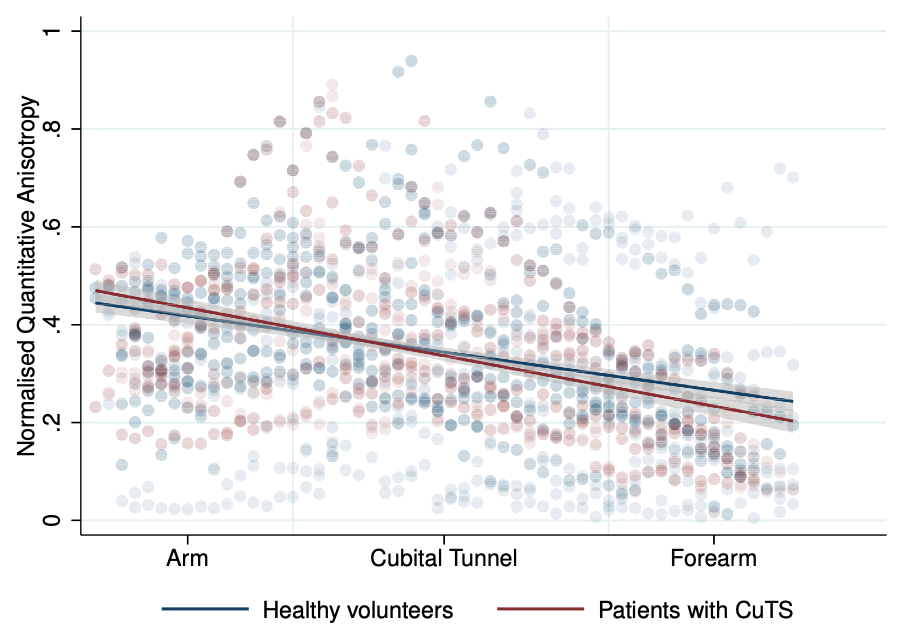


**eFigure 5.** Scatter plot with linear fit (and 95% CI) showing the positive correlation between normalised quantitative anisotropy and SNR.


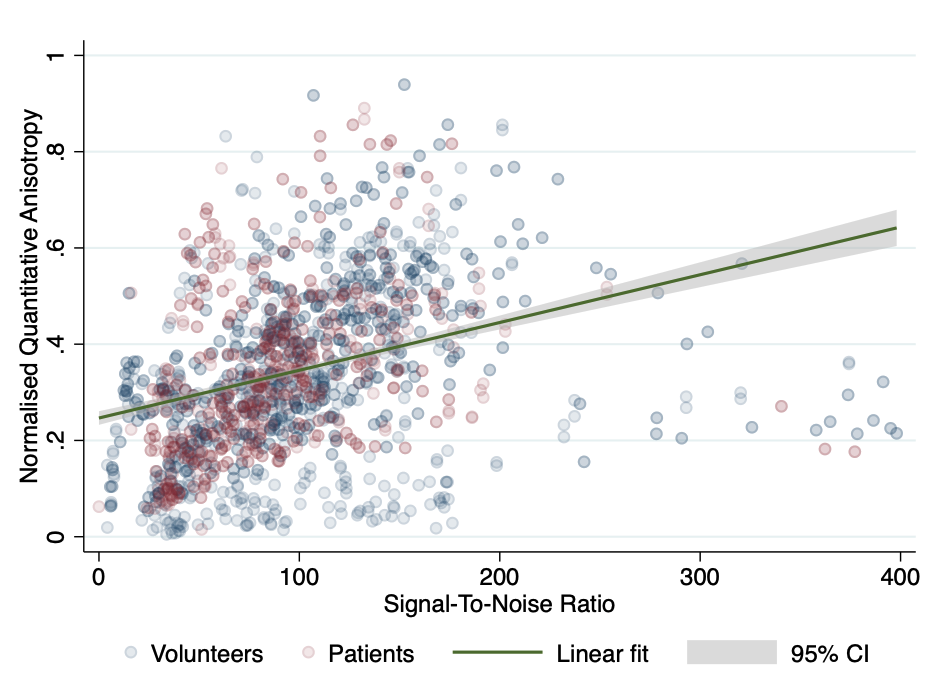


**eFigure 6.** Scatter plot with linear fit (and 95% CI) showing the association between normalised quantitative anisotropy and age.


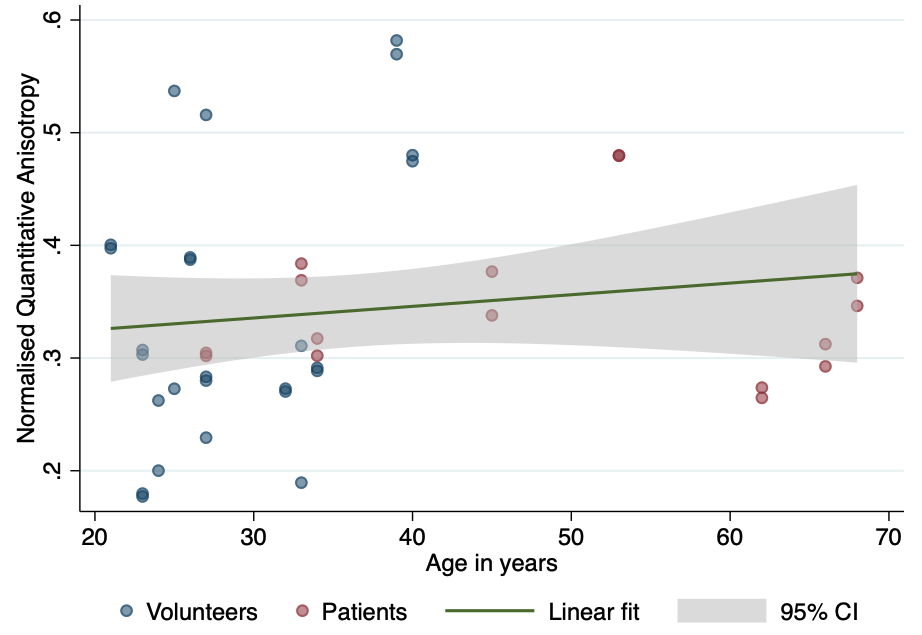


**eFigure 7.** Scatter plot with linear fit (and 95% CI) showing the relationship between mean diffusivity of the ulnar nerve in volunteers and patients, at different positions within the upper limb.


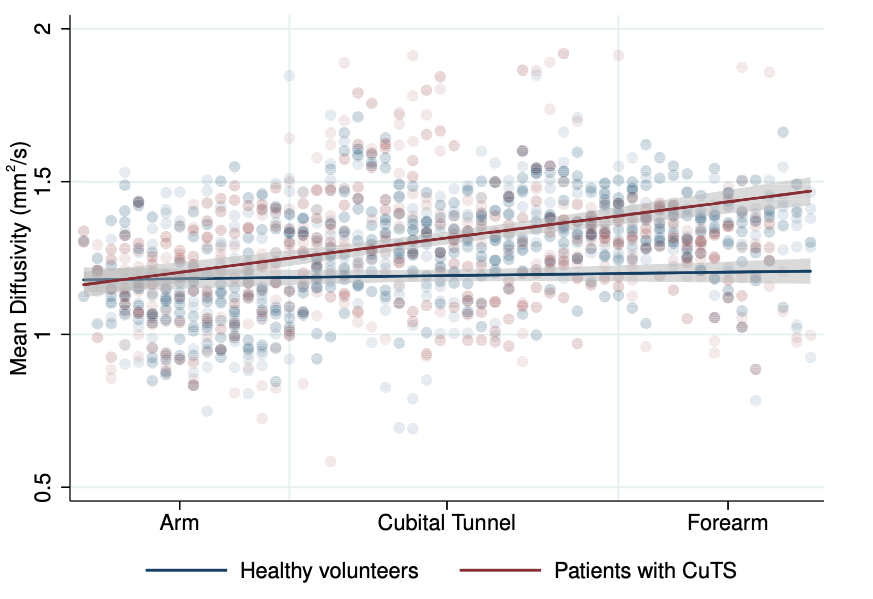


**eFigure 8.** Scatter plot with linear fit (and 95% CI) showing the association between mean diffusivity and SNR.

**
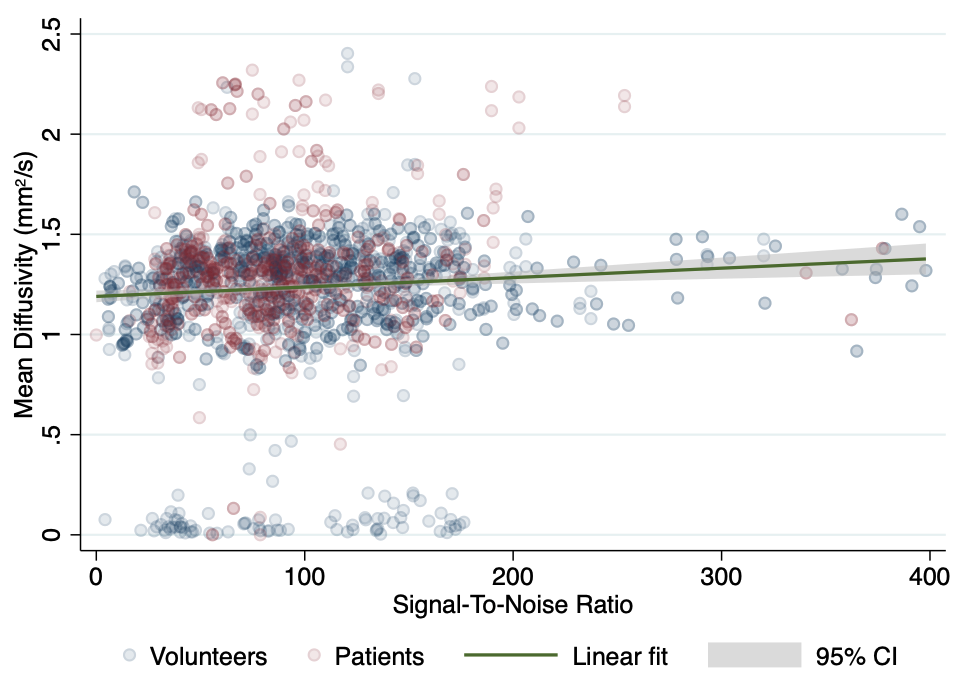
**

**eFigure 9.** Scatter plot with linear fit (and 95% CI) showing the association between mean diffusivity and age.


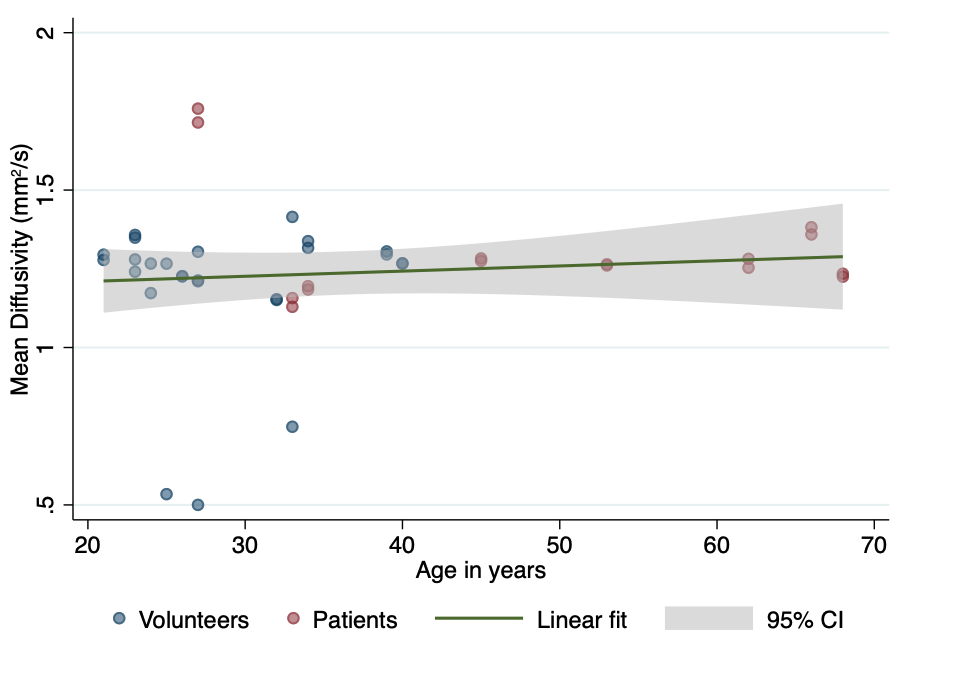


**eFigure 10.** Scatter plot with linear fit (and 95% CI) showing the relationship between axial diffusivity of the ulnar nerve in volunteers and patients, at different positions within the upper limb.


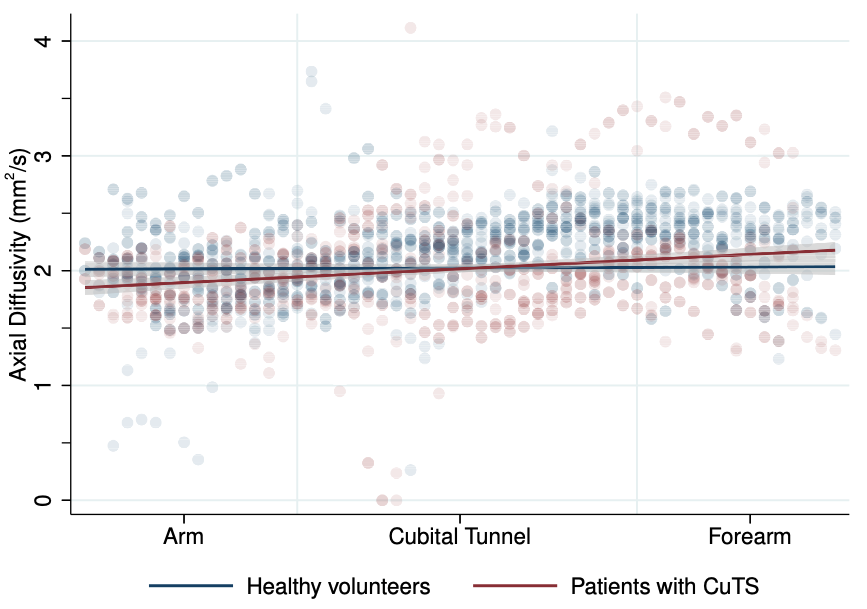


**eFigure 11.** Scatter plot with linear fit (and 95% CI) showing the association between axial diffusivity and age.

**
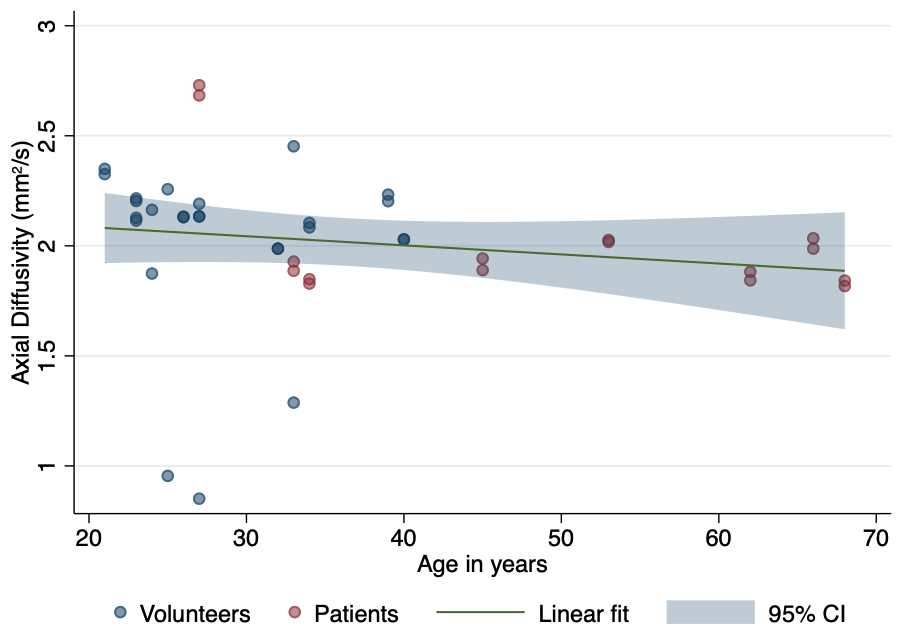
**

**eFigure 12.** Scatter plot with linear fit (and 95% CI) showing the association between axial diffusivity and SNR.

**
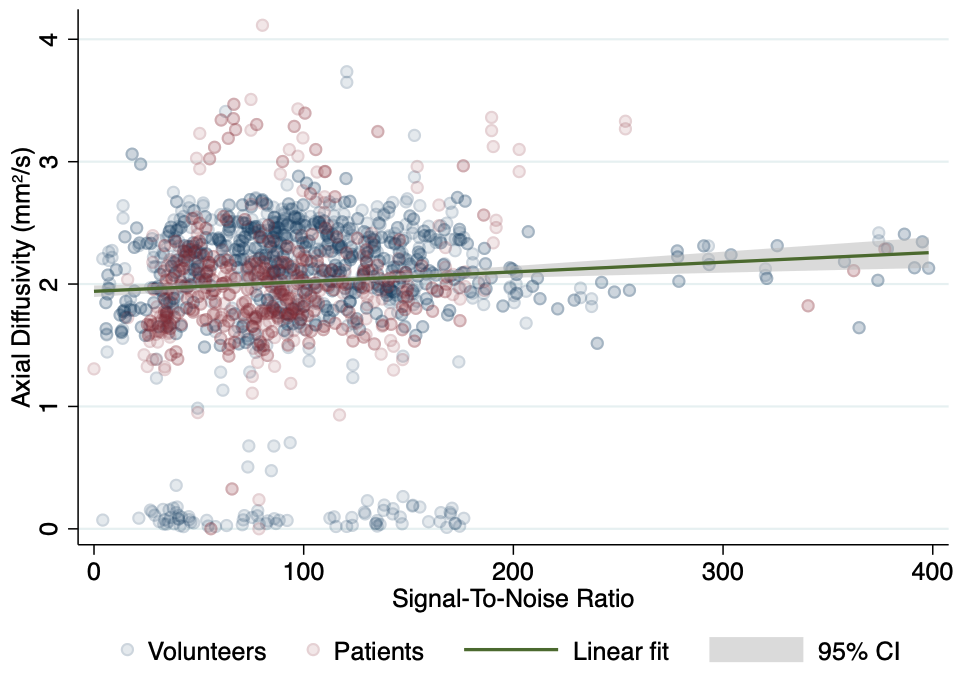
**

**eFigure 13.** Bland-Altman plot of the measured FA against the variability between raters. There is a cluster of disagreement in areas of low anisotropy.


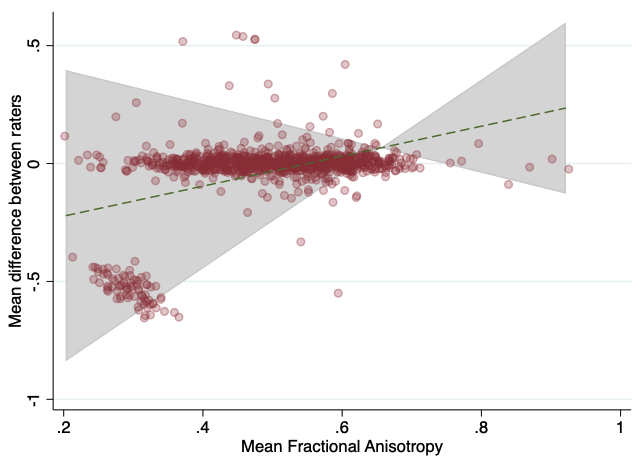

Supplement: Supplementary file 1 — Supplementary Figures. [file 41598_2021_94211_MOESM1_ESM.docx]
